# The Role of Heat Acclimation in Thermotolerance of Chickpea Cultivars: Changes in Photochemical and Biochemical Responses

**DOI:** 10.3390/life13010233

**Published:** 2023-01-13

**Authors:** Özlem Arslan

**Affiliations:** Department of Food Processing, University College of Espiye, University of Giresun, 28600 Giresun, Turkey; ozlem.turan@giresun.edu.tr

**Keywords:** *Cicer arietinum* L., chlorophyll *a* fluorescence transient, physiological and biochemical traits, high temperature

## Abstract

This study was conducted to determine the effects of heat stress on the physiological and biochemical responses of chickpea (*Cicer arietinum* L.; Diyar and Küsmen-99) cultivars that are both heat acclimated and non-acclimated. The seedlings were grown in soil for 15 days and then exposed to heat stress (35 °C, 5 days) after heat acclimation (30 °C, 2 days) or non-acclimation (25 °C, 2 days). Chlorophyll *a* fluorescence (ChlF) measurements were analyzed using the JIP test. Heat acclimation had no significant effect on ChlF parameters. Seedlings exposed to higher temperatures by acclimation were more tolerant in terms of ChlF parameters and Diyar had a better photochemical activity of photosystem II (PSII). Heat stress resulted in a decrease in electron transport efficiency, quantum yield, photosynthetic performance, and driving force in both chickpea cultivars, while K-band, L-band, and quantum yield of dissipation increased, especially in the non-acclimated cultivars. Additionally, ion leakage (RLR), malondialdehyde (MDA) content, and H_2_O_2_ synthesis increased in the cultivars, while water content (RWC), chlorophyll (*a* + *b*) content, and carotenoid content of the cultivars decreased. On the other hand, the cultivars attempted to eliminate reactive oxygen species (ROS) by increasing the content of anthocyanins and flavonoids and the activity of antioxidant enzymes (SOD and POD) under heat stress. Heat acclimation alleviated the negative effects of heat stress on each cultivar’s water content, chlorophyll and carotenoid content, membrane damage, photosynthetic activity, and antioxidant defense systems. The results of this study showed that, by providing heat acclimation more effectively, Diyar was better able to cope with the biochemical and physiological alterations that could be resulted from heat stress.

## 1. Introduction

Climate change increases the generation and dispersion of abiotic stresses that pose a serious risk to crop production [1]. Heat is an abiotic stress factor that limits plant development and crop yield. Heat stress is described as a temperature increase that exceeds a particular level over a period of time and irreversibly damages plant growth [2]. A temporary temperature rise of 10–15 °C above ambient temperatures is evaluated as heat stress [3]. When plants are exposed to heat stress, it inhibits plant growth and production by causing physiological and biochemical disorders in plants [4]. Heat stress leads to the denaturation and aggregation of proteins [2], disruption of membrane structures [5], inhibition of photosynthesis [6], deterioration of photosynthetic pigments [7], and alterations in antioxidant enzymes [8]. The main reason for these adverse effects is the negative effect of heat stress on photosynthetic activity. Photosystem II (PSII) is the most heat sensitive in the photosynthetic apparatus, and PSII activity is significantly reduced under heat stress [9]. Chlorophyll *a* fluorescence (ChlF) transients (OJIP), which can be used to determine the extent of photosynthetic responses of plants to heat stress, are a reliable, non-invasive and powerful tool for assessing photosynthetic electron transport. The signals recorded by ChlF allow the determination of the physiological state of plants, calculation of specific biophysical parameters, quantum yields, and probabilities that determine changes in PSII units, electron transport chain, and photochemical reactions by light [10,11,12,13,14]. Analysis of ChlF has been widely used in numerous studies to investigate various plant responses under heat stress, including rice [15], alfalfa [9], exotic weeds [16], tall fescue [7], barley [6], and maize [3]. The imbalance between the absorption and consumption of light energy due to heat stress leads to overexcitation of thylakoid membranes, resulting in photoinhibition. Heat stress leads to excessive energy loading of thylakoid membranes and eventually photoinhibition due to the imbalance between light energy absorption and utilization [17]. Photoinhibition is mainly due to the overproduction and accumulation of reactive oxygen species (ROS) such as hydroxyl radical (OH^−^), superoxide radical (O_2_^−^), and hydrogen peroxide (H_2_O_2_) [18]. Subsequently, the presence of excessive amounts of ROS leads to oxidative stress and oxidative stress damages all cellular structures, especially membranes [9]. To alleviate the ROS-induced oxidative injury, plants generate antioxidant defense systems (enzymatic and non-enzymatic) to scavenge the overproduced ROS [19]. The enzymatic antioxidant defense system includes several antioxidant enzymes: superoxide dismutase (SOD), peroxidase (POD), ascorbate peroxidase (APX), catalase, etc. Non-enzymatic antioxidants include the metabolites: ascorbate, carotenoids, anthocyanins, flavonoids, etc. The antioxidant defense mechanism in plants is part of the adaptation to heat and its strength correlates with the acquisition of thermotolerance. Thermotolerance can be achieved by heat acclimation with exposure to a non-lethal heat treatment [5]. Heat acclimation is increased tolerance to the physical and physiochemical exceedances of heat stress. This complex process, which involves physiological and biochemical alterations in plants, including rearrangements in the lipid composition of membranes, changes in the content of compatible metabolites, synthesis and accumulation of antioxidants and protective proteins, changes in hormone levels, and modifications of gene expression [20,21]. Even when heat acclimation is successful, plant susceptibility to heat stress varies with plant genotype and developmental stage; however, susceptibility is largely affected by genotype and species variability, as well as mostly intra- and inter-species variations [22].

Chickpea is a heat-sensitive cool season legume, as its potential yield decreases at temperatures above 35 °C [8]. The main growing areas of chickpea are in the arid and semi-arid zones of the world and due to climate change, it will be inevitable that the potential yield of chickpea will decrease due to the increase in the intensity and duration of exposure to high temperatures. Since the chickpea is an economically and agriculturally valuable crop, it was very important to investigate the responses of this crop to heat stress and heat acclimation, which our research group had previously studied under chilling [23,24], freezing [25,26] and drought conditions [26,27]. Karacan et al. [26] studied 18 chickpea cultivars using a multi-criteria decision making method to rank them according to their cumulative tolerance to cold and drought stress conditions, using physiological and biochemical analysis data from previous studies. According to the research results, when chickpea cultivars were ranked according to these two stress responses, Diyar scored quite differently from the other cultivars and was classified as tolerant, while Küsmen-99 was classified as moderately tolerant with an average score. Therefore, the heat stress responses of these two cultivars, classified as drought and cold tolerant (Diyar) and moderately tolerant (Küsmen-99), were investigated. To this end, two chickpea cultivars (Diyar and Küsmen-99) were subjected to heat stress (35 °C for 5 days) with or without heat acclimation (30 °C for 2 days) to understand the interaction between heat tolerance and heat acclimation on PSII photochemical activity, pigments, membrane stability, and defense mechanisms. The objective of this study was to (1) elucidate the physiological mechanisms, especially the photochemical activity of PSII and antioxidant defense systems in chickpea under heat stress; (2) explain the mitigating effects of heat acclimation on the mechanisms damaged by heat stress; (3) compare the thermotolerance of the cultivars studied; (4) determine the role of the correlation between oxidative stress and endogenous defense systems in the thermotolerance of the cultivars.

## 2. Materials and Methods

Seeds of chickpea (*Cicer arietinum* L.) cultivars (Diyar and Küsmen-99) were obtained from the Central Research Institute of Field Crops in Ankara, Turkey. To prevent fungal infections, to which chickpea is frequently exposed, seeds were treated with pesticides [Benomyl and Thriam (0.3 g per 100 g of seed)] and were sown in pots (3 seeds each) containing 325 g of air-dried soil. The soil had the following characteristics: Texture, clay [28]; water holding capacity, 20.1% [29]; pH, 7.54 [30]; EC, 258 µS cm^−1^ [31]; N, 1.48 g kg^−1^ [32]; P, 16.25 mg kg^−1^ [33]; and K, 464 mg kg^−1^ [33]. 100 μg g^−1^ NH_4_NO_3_ and 100 μg g^−1^ KH_2_PO_4_ were added to the soil, because the N, P, and K levels were found to be insufficient for chickpea. Plants were grown for 15 days in a growth chamber under good irrigation, at 25 ± 1 °C/20 ± 1 °C (day/night), a 16/8 h (day/night) photoperiod, a relative humidity of 60 ± 5%, and a light intensity of 250 μmol m^−2^ s^−1^ and then randomly divided into the following groups to conduct the experiments:

C_0_ and C, 17- and 22-day-old control seedlings grown under control conditions (25 ± 1 °C/20 ± 1 °C);

A, 17-day old heat-acclimated seedlings (grown under control conditions for 15 days, then exposed to 30 ± 1 °C/25 ± 1 °C for 2 days);

A + S, 22-day-old heat-treated acclimated seedlings (heat-acclimated and then exposed to 35 ± 1 °C/30 ± 1 °C for 5 days);

S, 22-day-old heat-treated non-acclimated seedlings (grown for 17 days under control conditions, then exposed to 35 ± 1 °C/30 ± 1 °C for 5 days).

The central leaves of the seedlings were used for the experimental analyses.

Since no statistically significant difference was found between the 17- and 22-day-old control groups (C_0_ and C) in all physiological and biochemical analyses examined, the results of the study were evaluated using the 22-day-old control group (C).

### 2.1. Polyphasic Chlorophyll a Fluorescence (ChlF) Measurement

ChlF transients were determined in dark-adapted leaves (6 replicates) using a Handy PEA fluorimeter (Plant Efficiency Analyser, Hansatech Instruments Ltd., Norfolk, UK). After a 30-min dark adaptation, leaves were irradiated with light (3000 μmol m^−2^ s^−1^) for one second and the intensity of fluorescence at 20 µs (F_0_), 300 µs (F_K_), 2 ms (F_J_), 30 ms (F_I_), and maximum fluorescence (F_P_) were determined [10]. The JIP test parameters were calculated from obtained fluorescence intensities. The effects of heat stress on cultivars were assessed based on relative fluorescence between the steps O and K [20 and 300 μs, respectively = V_OK_ = (F_t_ − F_0_)/(F_K_ − F_0_)], O and J [20 μs and 2 ms, respectively = V_OJ_ = (F_t_ − F_0_)/(F_J_ − F_0_)] and I and P [30 ms and at the peak P of OJIP, respectively = V_IP_ = (F_t_ − F_I_)/(F_P_ − F_I_)] were normalized and given as the kinetic difference V_OK_ = V_OK(treatment)_ − V_OK(control)_, V_OJ_ = V_OJ(treatment)_ − V_OJ(control)_ and V_IP_ = V_IP(treatment)_ − V_IP(control)_, respectively [10,11]. The efficiencies and quantum yields of fluorescence were also calculated: φ_P0_, (1 − F_0_/F_M_ or F_V_/F_M_), maximum quantum yield of primary photochemistry; ψ_E0_, (1 − V_J_), probability that a trapped exciton moves an electron into the electron transport chain beyond Q_A_^−^; φ_E0_, [(1 − F_0_/F_M_) × ψ_E0_], quantum yield for electron transport; φ_D0_, (DI_0_/ABS), quantum yield of energy dissipation; φ_R0_, (φ_P0_ × ψ_0_ × δ_R0_), the quantum yield of electron transport from Q_A_^−^ to the PSI end electron acceptors; δ_R0_, (1 − V_I_)/(1 − V_J_), the efficiency with which an electron can move from the reduced intersystem electron acceptors to the PSI end final electron acceptors. The performance indexes (PI_ABS_ and PI_TOTAL_) were calculated from the components to determine the difference between the cultivars PI_ABS_, [(RC/ABS) − [φ_P0_/(1 φ_P0_)] [ψ_0_/(1 − ψ_0_)], performance index (potential) for energy conservation from photons absorbed by PSII to the reduction of intersystem electron acceptors; PI_TOTAL_, PI_ABS_ [(δ_R0_/(1 − δ_R0_)], performance index (potential) for energy conservation from photons absorbed by PSII to the reduction of PSI end acceptors; DF, log(PI_ABS_), driving force on absorption basis [10,11].

### 2.2. Water Content and Pigment Analysis

To determine the percent relative water content (RWC) of leaf segments (R = 0.5 cm and 6 replicates), fresh leaves were weighed (FW) and then incubated in 10 mL distilled water for 24 h to determine the saturated weight (SW), and the leaves were dried at 80 for 48 h, their dry weight (DW) was determined, and the RWC was calculated as (%) = [(FW − DW)/(SW − DW)] × 100 [34]. After extraction of the leaves (0.1 g with 6 replicates) in 100% acetone, they were measured spectrophotometrically (at wavelengths 470, 644.8, and 661.6 nm), and the content of chlorophyll (Chl) (*a* + *b*) and carotenoids (*x* + *c*) (mg g^−1^ FW) was calculated [35]. To determine anthocyanin content (mg g^−1^ FW) and flavonoid content (%), fresh leaf samples (0.1 g with 3 replicates) were ground in acidified methanol [methanol:water:HCl (79:20:1)] and measured at wavelengths of 530 and 657 nm for anthocyanin and 300 nm for flavonoid, respectively. Anthocyanin was calculated according to the method of Mancinelli et al. [36]. Flavonoid was calculated as a percentage of the content of 22-day-old control plants (C) [37].

### 2.3. Relative Leakage Ratio, MDA, and H_2_O_2_ Contents

Relative leakage ratio (RLR) was measured indirectly as leakage of UV-absorbing substances according to the method of Redmann et al. [38]. Five leaf segments (R = 0.5 cm) with three replicates were kept in 10 mL distilled water for 24 h and measured at 280 nm (A_1_). Samples were treated in liquid nitrogen and shaken for another 24 h in incubation water. The samples were measured at 280 nm (A_2_) and the RLR was calculated as A_1_/A_2_ [38]. Malondialdehyde (MDA) content (nmol g^−1^ FW) was determined (0.1 g leaf samples with 3 replicates) as described by Hodges et al. [39] and MDA was calculated using the extinction coefficient (157 mM^−1^ cm^−1^). To determine the amount of H_2_O_2_ (nmol g^−1^ FW), leaf samples (0.1 g and 3 replicates) were extracted in 0.1% TCA with 0.1 M Tris-HCl (pH 7.6). The extracts were treated with potassium iodide reagent and kept in the dark for 90 min. Samples were measured at 390 nm and calculated using the standard curve [40].

### 2.4. Antioxidant Enzyme Activities

Soluble protein was extracted from leaves (0.5 g with 3 replicates) to determine the enzyme activities. The Bradford method [41] was used to determine the protein concentration and the leaf samples were extracted in the corresponding extraction buffer. 1 mL of buffer solution (9 mM Tris-HCl and 13.6% glycerol) was added to the powdered samples with liquid nitrogen and the total SOD activity (EC 1.15.1.1) (U mg protein^−1^) was determined [42]. The buffer solution of the leaves homogenized for POD (EC 1.11.1.7) and CAT (EC 1.11.1.6) included 100 mM potassium phosphate buffer (pH 7.0), 2% PVP, and 1 mM Na_2_EDTA. The POD activity was determined by measuring the oxidation of guaiacol (ɛ = 26.6 mM cm^−1^) by H_2_O_2_ (nmol H_2_O_2_ min^−1^ mg protein^−1^) at 470 nm [43]. The CAT activity was calculated as nmol H_2_O_2_ min^−1^ mg protein^−1^, with the absorbance values at 240 nm decreasing according to the dissociation of H_2_O_2_ [44].

### 2.5. Statistical Analysis

The research experiments were conducted in a completely randomized design. The experiment was laid out in three replicates with 90 plants in a total of 30 pots, and mean values (*n* = 3 or 6) were obtained for each treatment. Analysis of variance (*ANOVA*) was performed for all data obtained from the experiments. The variability of data among cultivars and treatments was calculated using the least significant difference (LSD) test at the 95% probability level (*p* < 0.05). *SPSS* v 20.0 (Chicago, IL, USA) was used for all research data analyzes.

## 3. Results

### 3.1. Effect of Heat Stress on Chickpea ChlF Rise and ChlF Parameters

**Figure 1:**
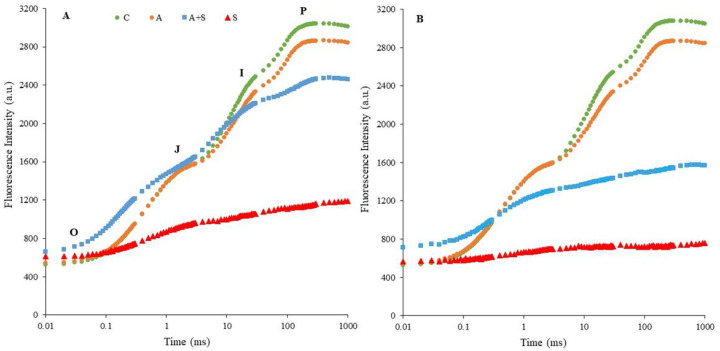
Induction curves of polyphasic ChlF in chickpea cultivars ((**A**,**B**), Diyar and Küsmen-99, respectively) exposed to heat stress with or without heat acclimation. The transients are plotted on a logarithmic time scale (10 μs to 1 s). The mean values of the OJIP transients are plotted, *n* = 6.

The OJIP transients measured as ChlF rises in dark-adapted control and stressed chickpea leaves were determined by plotting them on a logarithmic time scale (Figure 1). The OJIP rise reflects three reduction processes in the electron transport chain (O-J, J-I, and I-P phases) [6,10]. The O-J rise contains information about the antenna size and indicates the reduction on the acceptor side of PSII [45]. The J-I phase refers to the kinetic properties required for the reduction and/or oxidation of the plastoquinone pool (PQ) [46]. The I-P phase represents the re-reduction of plastocyanin and the acceptor side of PSI [6,46]. Exposure to 35 °C significantly altered the shape of the typical OJIP curves seen in controls. The reduction in fluorescence intensity was more pronounced in both heat-acclimated (A + S) and non-acclimated (S) treatments of Küsmen-99 (Figure 1B). The S treatment caused the disappearance of the J-I and I-P phases, while the P level approached the O-J phase, indicating photochemical inhibition of PSII. A similar effect was determined in the heat-acclimated stress treatment (A + S) of Küsmen-99.

**Figure 2:**
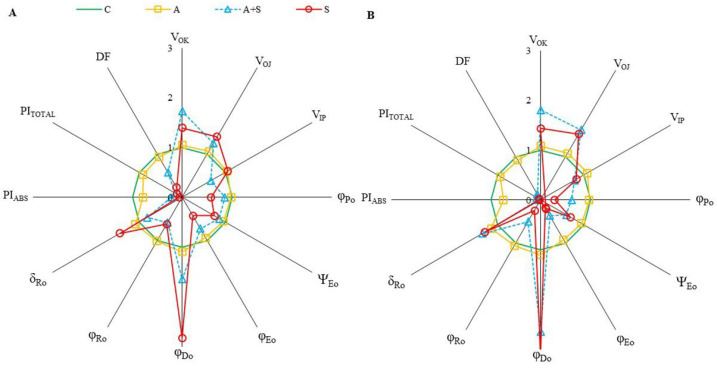
The radar-plot presentation of selected OJIP parameters in chickpea cultivars ((**A**,**B**), Diyar and Küsmen-99, respectively) exposed to heat stress with or without heat acclimation. The mean values of the parameters were plotted in relation to the corresponding controls, *n* = 6.

ChlF parameters, which provide information about photosynthetic fluxes and quantify the PSII and PSI behaviors are derived from ChlF transients. The parameters representing the relative values of controls were shown by spider plot graphics (Figure 2). Exposure to heat acclimation (30 °C for 2 days, A) resulted in slight changes in both cultivars compared to corresponding controls. However, significant changes in almost all selected ChlF parameters were determined in both cultivars exposed to heat stress (35 °C for 5 days), whether acclimated (A + S) or non-acclimated (S), compared to the controls. Heat stress resulted in a similar extent increase in both V_OK_ and V_OJ_ parameters in Diyar and Küsmen-99 (Figure 2A and Figure 2B, respectively). The V_OK_ and V_OJ_ parameters are expressed as L- and K-bands, respectively, and reflect the inactivation of the oxygen-evolving-complex (OEC). The V_IP_ values decreased when the cultivars were subjected to 35 °C, except S treatment of Diyar (Figure 2A). The decrease in V_IP_ values (G-band) indicates limitations in electron transport on the PSI acceptor side. The maximum quantum yield of the photochemistry of PSII (φ_P0_ = TR_0_/ABS = F_V_/F_M_) of chickpea cultivars reduced in both acclimated and non-acclimated heat stress treatments (Figure 2). The highest φ_P0_ decreases were determined in A + S (38%) and S (72%) treatments of Küsmen-99 (Figure 2B). The parameter ψ_E0_ (ET_0_/TR_0_) explains the probability that captured exciton moves the electron further in the electron transport chain than Q_A_^−^. The highest decreases in ψ_E0_ values were determined in Küsmen-99 during heat stress, especially heat acclimated (41% of control). The φ_E0_ value that defines the quantum yield efficiency that captured exciton moves electron to the electron transport chain (φ_E0_ = ET_0_/ABS), declined markedly in all cultivars due to heat stress treatments, and the highest decline of φ_E0_ results was determined in A + S (63%) and S (81%) treatments of Küsmen-99 (Figure 2B). Heat treatments led to marked increases in the quantum yield of dissipation (φ_D0_ = DI_0_/ABS) values of both cultivars (Figure 2). Küsmen-99 exhibited the highest increment of A + S and S treatments 2.6- and 3.1-fold of control, respectively. Heat reduced quantum yield of electron transport from Q_A_^−^ to the PSI end electron acceptors (φ_R0_ = RE_0_/ABS) values in cultivars, mainly in S treatment of Küsmen-99 (76%). The parameter δ_R0_ (RE_0_/ET_0_), which reflected the probability that electron was transferred from intersystem electron carried to electron acceptors at PSI acceptor side was significantly increased by heat stress treatments in all cultivars, except 19% decrease in the A + S treatment of Diyar (Figure 2). The cultivars exhibited a gradual decrease in the values of performance indexes (PI_ABS_ and PI_TOTAL_) in both heat acclimation and heat stress treatments (Figure 2). In determining PSII behavior, PI_ABS_ refers to energy absorption, capture, and conversion in electron transport steps. Heat acclimation led to a significant decrease in PI_ABS_ of both Diyar and Küsmen-99 (21% and 25%, respectively). Additionally, the highest reduction was determined in non-acclimated heat stress treatment of the cultivars, Diyar (94%) and Küsmen-99 (98%). The PI_TOTAL_ parameter includes additional electron steps to PI_ABS_, and PSI refers to the measure for performance up to the reduction of final electron acceptors. The extent of the reductions of the PI_TOTAL_ was remarkable in both A + S and S treatments for Diyar (84% and 87%, respectively) and Küsmen-99 (91% and 96%, respectively). Likewise, the PI_ABS_ and PI_TOTAL_, the total driving force for photosynthesis (DF = log PI_ABS_) values of cultivars declined gradually with heat stress treatments (Figure 2). Among the cultivars, Küsmen-99 had the highest reductions, especially for the S treatment (5-fold of the corresponding control).

### 3.2. Effect of Heat Stress on Chickpea Water and Pigment Contents

The relative water content (RWC) of the leaves of the cultivars declined sharply in all heat treatments, including heat acclimation (Diyar and Küsmen-99, 9% and 20%, respectively) (Table 1). Exposure to 35 °C with heat acclimation (A + S) resulted in significant reductions (Diyar and Küsmen-99, 35% and 46%, respectively), while the non-acclimated heat stress treatment (S) resulted in the highest reductions (Diyar and Küsmen-99, 41% and 57%, respectively). All heat treatments significantly reduced the Chl (*a* + *b*) content of the cultivars (Table 1). The extent of Chl (*a* + *b*) reduction caused by heat acclimation was not as great as that by heat stress treatments. Heat acclimation led 17% and 12% reduction in control levels for Diyar and Küsmen-99, respectively. In addition, the magnitude of the reduction in Chl (*a* + *b*) content for the A + S and S treatments was 23% and 33%, respectively, for Diyar and 48% and 56%, respectively, for Küsmen-99. Similarly, all treatments resulted in a gradual decrease in the carotenoid content of the cultivars. The A + S treatment resulted in a 47% and 57% reduction in carotenoid content of Diyar and Küsmen-99, respectively, with the highest reduction determined in the S treatment of Diyar (60%) and Küsmen-99 (66%). In contrast to the results for Chl (*a* + *b*) and carotenoids, anthocyanin and flavonoid contents of cultivars subjected to heat treatments significantly increased (Table 1). The increase in anthocyanin content was more pronounced in all treatments (A, A + S, and S) of Diyar (3.3-, 5.7- and 4.9-fold of the corresponding control, respectively), while the highest flavonoid content was determined in the heat treatments (A + S and S) of Küsmen-99 (87% and 85%, respectively).

### 3.3. Effect of Heat Stress on Chickpea Membrane Integrity and Lipid Peroxidation

**Figure 3:**
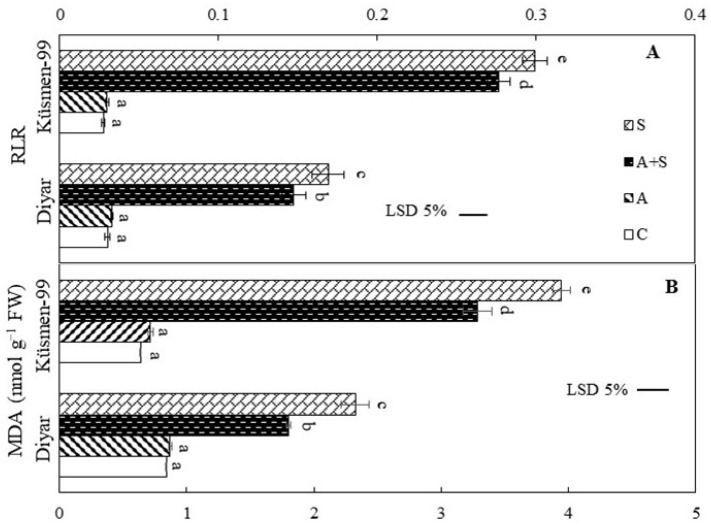
Heat stress with or without heat acclimation resulted in changes in RLR (**A**) and MDA contents (**B**) in chickpea cultivars. The values are presented as the mean ± standard error (SE), *n* = 3. The bars and different letters indicate significant differences between treatments and cultivars at *p* < 0.05 according to the LSD test.

The heat acclimation period did not cause any membrane damage in the leaves of cultivars according to the relative leakage ratio (RLR) and malondialdehyde (MDA) results (Figure 3). Heat treatments, both heat acclimated and non-acclimated, led to a dramatic increase in the RLR, indicating loss of membrane integrity in the leaf cells of chickpea cultivars (Figure 3A). RLR increased 4.9- to 5.6-fold in Diyar and 10- to 10.9-fold in Küsmen-99 under A + S and S treatments, respectively. Similar results were obtained for MDA contents that reflect the lipid peroxidation of cellular membranes. The MDA levels increased 2.1- and 2.8-fold in heat treatments (A + S and S, respectively) in Diyar and 5.1- and 6.2-fold in Küsmen-99 compared with control (Figure 3B).

### 3.4. Effect of Heat Stress on Chickpea H_2_O_2_ Content and Antioxidant Enzyme Activities

**Figure 4:**
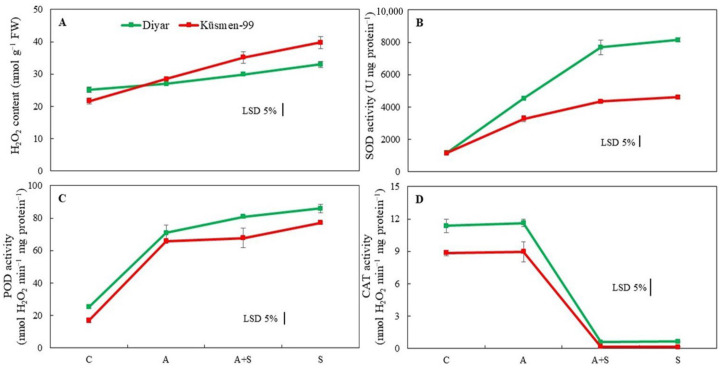
Heat stress with or without heat acclimation induced changes in H_2_O_2_ content (**A**) and antioxidant enzyme activities (SOD, (**B**); POD, (**C**) and CAT, (**D**)) of chickpea cultivars. The values are presented as the mean ± standard error (SE), *n* = 3. The bars indicate significant differences between treatments and cultivars at *p* < 0.05 according to the LSD test.

The H_2_O_2_ content of the cultivars increased during heat stress treatments, indicating oxidative stress (Figure 4A). While the increase during heat acclimation (A) treatment was not significant in the Diyar cultivar, A treatment caused a 32% increase in Küsmen-99. Moreover, heat stress treatments caused a gradual increase in the H_2_O_2_ content of Diyar and Küsmen-99, especially in S treatment, by 31% and 84%, respectively. Superoxide dismutase (SOD) activity increased markedly in all heat-treated (A, A + S, and S) chickpea cultivars, although it was more pronounced in Diyar (3.9-, 6.7-, and 7.1-fold, respectively, of the corresponding control) (Figure 4B). Similar to SOD, all heat treatments resulted in a significant increase in peroxidase activity (POD) of both cultivars compared to the corresponding controls (Figure 4C). However, the increase in activity between A + S and S treatments in Diyar and A and A + S treatments in Küsmen-99 proved to be insignificant compared to each other. In contrast to SOD and POD, the catalase (CAT) activity of cultivars declined sharply in heat stress treatments, regardless of whether they were acclimated or not, while heat acclimation (A) treatment did not cause any significant change in CAT activity (Figure 4D). However, when acclimated and non-acclimated heat treatments (A + S and S) were compared, no significant differences in the CAT activity were determined for either cultivar.

## 4. Discussion

The objective of the study was to elucidate the physiological and biochemical mechanisms involved in the tolerance of chickpea cultivars to heat stress, either acclimated or non-acclimated. Plants develop different tolerance mechanisms to overcome the deleterious effects of high-temperature stress, especially when acclimated to heat [5]. The effects of heat acclimation on the biochemical and physiological mechanisms of two *Cicer arietinum* L. cultivars subsequently exposed to higher temperatures were studied. Photosynthetic responses to rising temperatures play a critical role in regulating plant heat tolerance. One of the most important responses in regulating plant heat tolerance is the photosynthetic response that the plant develops as temperatures rise. The most heat-sensitive components of the electron transfer chain are the units responsible for photosynthesis and, in particular, the oxygen-evolving complex (OEC) of PSII [6]. Heat stress inhibits photosynthesis by altering the redox balance of electron transport reactions [6,47]. In this study, chickpea cultivars exhibited higher F_O_ values, especially when heat acclimated at 35 °C. Elevated F_O_ values represent increased damage to chloroplasts due to heat stress, resulting in the inhibition of energy transfer to PSII and reduced quantum efficiency of PSII [22]. Fluorescence densities F_J_, F_I_, and F_P_ also decreased in phases J, I, and P of cultivars exposed to heat stress. These changes reflect the inhibition of electron transport from the OEC to the PQ pool. The curves show that the I and P phases decreased similarly in both cultivars during heat acclimation (A). In addition, significant differences were determined between the cultivars in both heat stress treatments when the ChlF curves were examined. Non-acclimated heat treatments (S) in both cultivars and acclimated stress treatment of Küsmen-99 made the OJIP curves to disappear on the logarithmic scale. Remarkably, the effects of heat stress on the OJIP curve were much more pronounced in the S treatments. The results show that OEC and reduction/reoxidation of Q_A_ and Q_B_ are more susceptible to high temperatures in Küsmen-99 than in Diyar. Similar effects of high temperature on photoinhibition of PSII have been reported previously [15,48]. The differential effects of heat on PSII could be due to different cyclic electron flow capacities around PSI [15], which could result from the genetic variation of cultivars. Heat stress treatment resulted in an increase in V_OK_ and V_OJ_, indicating the alteration of L- and K- bands, respectively. The presence of the L-band provides information about the utilization of excitation energy, while the K-band refers to the stable electron transfer from OEC to P680^+^ and subsequently to Q_A_^−^ [10,12]. Higher K-band is a heat indicator to predict plant response to heat [16]. Küsmen-99 showed higher L- and K-bands than Diyar in acclimated and non-acclimated heat treatments. It has been reported that an increased L-band indicates a loss of connectivity between the reaction centers and their antenna complexes, while elevated K-band value represents an inhibition of OEC due to Mn-complex injury [6,10]. In contrast to the results of V_OK_ and V_OJ_, the levels of V_IP_ decreased in all treatments, except non-acclimated Diyar. The V_IP_ indicates the changes in the G-band related to electron transfer from PSII to PSI [13]. Thus, these bands (K-, L-, and G-) formed as a result of the heat stress showed that the light reactions of photosynthesis, particularly the acceptor side of PSII were negatively affected. Moreover, heat stress markedly changed the efficiency and quantum yield of PSII *(*φ_P0_, ψ_E0_, φ_E0_, φ_D0_, φ_R0_, and δ_R0_) of chickpea cultivars. Among the efficiency and quantum yield parameters, φ_P0_, ψ_E0_, φ_E0_, and φ_R0_ were lower, whereas φ_D0_ and δ_R0_ were higher in both acclimated and non-acclimated heat treatments. The only exception was the δ_R0_ parameter in the acclimated heat treatment of Diyar. The increased values of φ_D0_ indicated that the trapped energy was probably radiated as heat energy and the connection between the photosynthetic systems was broken [11]. The results showed that electron transfer from PSII was inhibited by heat on both the electron donor and acceptor sides. All heat treatments caused photoinhibition in both cultivars and photoinhibition of PSII was alleviated by heat acclimation. Chickpea cultivars exposed to heat treatments exhibited reductions in both PI_ABS_ and PI_TOTAL_. Changes in the efficiencies and quantum yields of the photosystem could be the reason for alterations in the performance indexes, which are multiparametric expressions for successive steps in primary photochemical reactions. While PI_ABS_ describes the part up to the reduction of intersystem electron transport of photons absorbed by the PSII reaction centers, PI_TOTAL_ describes the part up to the reduction of the PSI final electron acceptor [10,12]. Reductions in performance indexes and DF indicate impairment of the photochemical activities of the reaction centers. The main reason for these disruptions in photochemical activities as a result of heat stress is oxidative stress, which results from the increased formation of ROS in the thylakoid membranes [49].

Heat stress causes wilting, curling and yellowing of leaves as well as a reduction in plant biomass, suggesting that heat stress causes plants to reduce growth and trigger stomatal closure to prevent water loss [4,22]. RWC gradually declined under heat-stress temperatures. Previous reports showed that heat-tolerant wheat [50] and alfalfa [22] cultivars had the highest water content. Since Diyar had higher RWC values, the cultivar could be classified as heat tolerant. Treatments with heat acclimation had higher RWC values than treatments without acclimation, suggesting that the heat acclimation period may play an important role in maintaining the homeostasis of heated cells. When plants are exposed to any stress, this stress is accompanied by oxidative stress. It is well known that chloroplasts are the main source of ROS generation under stress conditions due to the limitation of electron transport [51]. Since chlorophyll is a necessary pigment for photosynthesis, the varying amount of total chlorophyll is a decisive indicator of the level of photosynthesis in plants [4]. The Chl (*a* + *b*) content of cultivars was drastically reduced by heat temperature, regardless of whether acclimated or non, especially in Küsmen-99. Similar results occurred in chickpea plants under heat stress, and this damage to pigment was found to be due to the photooxidation of chlorophyll [8]. Carotenoids, non-enzymatic antioxidants, protect chlorophyll from photooxidation [3]. In this study, the carotenoid content of the cultivars declined in all heat treatments. It was found that the content of photosynthetic pigments was higher in heat-tolerant chickpea genotypes than in the other genotypes [52]. Since Diyar always contains more chlorophyll and carotenoids in heat treatments, the results of this study are consistent with the literature. Contrary to chlorophyll and carotenoids, the increased levels of anthocyanins and flavonoids were determined in heat treatments. Anthocyanins and flavonoids act as antioxidants in plants and maintain chloroplast functionality by protecting chlorophyll from photoinhibition under heat-stress conditions. However, the reduced photosynthetic efficiency of chickpea under heat treatment indicated that the light screening role of anthocyanins and the antioxidant roles of flavonoids was not sufficient to prevent the overexcitation of chloroplasts, especially at Küsmen-99.

Heat stress leads to lipid peroxidation of cell membranes and membrane injury in numerous plants [5,19,22,53]. The heat stress treatments led to a marked increase in RLR and MDA results of the cultivars, but this increase remained lower increase due to heat acclimation. The increase in the RLR ratio indicates electrolyte leakage and loss of membrane stability. The excessive accumulation of MDA is due to the induction of lipid peroxidation in cell membranes by ROS, which is formed and accumulated by heat stress. Oxidative stress, which occurs as a result of increased ROS production and accumulation due to metabolic disorders, is known to be an important indicator of stress in plants. H_2_O_2_ is one of the ROS and is highly toxic to plant tissues [5]. Chickpea cultivars were found to have significantly increased H_2_O_2_ levels under heat stress. However, the increase in H_2_O_2_ levels was lower in acclimated cultivars than in non-acclimated cultivars. Although H_2_O_2_ is a stimulant to increase antioxidant capacity under stress conditions, its presence in cellular components above a threshold level is an indicator of oxidative stress [19]. Plants have antioxidant defense mechanisms to prevent excessive ROS production and improve tolerance to oxidative stress. It was found that plants with high levels of antioxidant enzymes also have a high tolerance to ROS-induced oxidative damage [4]. The results showed that both cultivars exhibited higher activity of antioxidants in all heat stress treatments, except CAT. Disturbances in the photochemical activity of PSII due to heat stress may lead to increased production of superoxide radicals (O_2_^−^), the secondary product of the electron transfer chain, and thus to an increase in the activity of SOD [54]. SOD plays a role as the first defense against ROS, by converting O_2_^−^ to O_2_ and H_2_O_2_ [55]. Subsequently, POD, one of the antioxidants that detoxify H_2_O_2_, removes H_2_O_2_ by oxidizing components such as phenolic compounds and/or antioxidants. In the present study, although both SOD and POD activities increased with heat stress, the elevated RLR, MDA, and H_2_O_2_ levels indicated insufficient to remove oxidative stress in cultivars. In addition, ascorbate peroxidase (APX) and glutathione reductase (GR) are enzymes of the Halliwell–Asada pathway, one of the metabolic systems responsible for the detoxification of H_2_O_2_. It was reported that the activities of APX and GR increased in maize exposed to high temperatures [56]. Moreover, heat tolerance at lethal temperatures was found to be associated with increased activities of SOD and APX [57]. In contrast to SOD and POD, the activity of CAT exhibited a pronounced decline under heat treatments. CAT degrades H_2_O_2_ to H_2_O and O_2_ and is primarily located in peroxisomes. The decrease in the activity of CAT under heat stress is due to the photoinactivation of catalase and decreased catalase synthesis. In addition, high H_2_O_2_ may lead to a decrease in the activity of CAT due to substrate inactivation. The reduced activity of CAT may contribute to H_2_O_2_ accumulation, which leads to lipid peroxidation under heat-stress conditions [53].

According to the research results, it was found that heat acclimation in chickpea cultivars increased heat tolerance at higher temperatures to which the cultivars were later exposed. The increased tolerance was found to be associated with the enhancement of protective mechanisms such as anthocyanins, flavonoids, and antioxidant enzymes. Acquisition of thermotolerance by prior heat acclimation reduced cellular leakage and membrane injury. Therefore, oxidative damage and heat injury was reduced in treatments subjected to heat acclimation. Tolerance differences among cultivars became more evident in seedlings exposed to heat stress, especially acclimation. Diyar, which is known to be cold and drought tolerant, responded similarly to heat stress as the acclimated Küsmen-99, although it was not acclimated in all parameters. However, heat tolerance of both cultivars increased significantly with acclimation. According to the polyphasic chlorophyll *a* fluorescence data, Diyar showed photosynthetic activity under heat stress that approached the control by acclimation, whereas Küsmen-99 did not improve photosynthetic activity. This could be due to the fact that Diyar is more successful in maintaining water, chlorophyll, and carotenoid content and increases its anthocyanin content under heat-stress conditions. In addition, the increase in antioxidant enzyme efficiencies while maintaining membrane damage and the lower H_2_O_2_ content are physiological changes that make Diyar more tolerant to heat stress. The cultivar Diyar was more successful than Küsmen-99 in coping with the negative effects of heat stress.

## Figures and Tables

**Table 1:** Relative water content (RWC) (%), chlorophyll (Chl) (*a* + *b*) (mg g^−1^ FW), carotenoid (mg g^−1^ FW), anthocyanin (mg g^−1^ FW), and flavonoid (%) content of chickpea cultivars subjected to heat treatments.

Cultivars	Treatment	RWC	Chl (*a* + *b*) Content	Carotenoid Content	Anthocyanin Content	Flavonoid Content
Diyar	C	65 ^1^ ± 1 ^a^	74 × 10^−3^ ± 0.0 ^a^	159 × 10^−4^ ± 0.0 ^a^	117 × 10^−6^ ± 0.0 ^a^	100 ± 0 ^a^
	A	59 ± 2 ^b^	62 × 10^−3^ ± 0.0 ^b^	119 × 10^−4^ ± 0.0 ^b^	387 × 10^−6^ ± 0.0 ^b^	111 ± 3 ^b^
	A + S	42 ± 1 ^c^	57 × 10^−3^ ± 0.0 ^c,f^	84 × 10^−4^ ± 0.0 ^c^	665 × 10^−6^ ± 0.0 ^c^	144 ± 8 ^c^
	S	38 ± 2 ^c,e^	50 × 10^−3^ ± 0.0 ^d^	64 × 10^−4^ ± 0.0 ^d^	574 × 10^−6^ ± 0.0 ^d^	152 ± 5 ^d^
Küsmen-99	C	67 ± 1 ^a^	67 × 10^−3^ ± 0.0 ^e^	141 × 10^−4^ ± 0.0 ^a^	259 × 10^−6^ ± 0.0 ^e^	100 ± 0 ^a^
	A	54 ± 1 ^d^	59 × 10^−3^ ± 0.0 ^f^	116 × 10^−4^ ± 0.0 ^b^	330 × 10^−6^ ± 0.0 ^f^	94 ± 1 ^e^
	A + S	36 ± 1 ^e^	35 × 10^−3^ ± 0.0 ^g^	61 × 10^−4^ ± 0.0 ^d,e^	442 × 10^−6^ ± 0.0 ^g^	187 ± 6 ^f^
	S	29 ± 1 ^f^	29 × 10^−3^ ± 0.0 ^h^	48 × 10^−4^ ± 0.0 ^e^	434 × 10^−6^ ± 0.0 ^g^	185 ± 3 ^g^
LSD 5%		4	3 × 10^−3^	13 × 10^−4^	55 × 10^−6^	

^1^ Each value is presented as the mean ± SEs, *n* = 6 (for RWC, Chl and carotenoid) or 3 (for anthocyanin and flavonoid). Different letters indicate significant differences between treatments and cultivars at *p* < 0.05 according to LSD 5%.

## Data Availability

All data are contained within the article.
